# Definitive Radiotherapy in Serous Carcinoma of Unknown Primary With Isolated Nodal Disease: A Case Review

**DOI:** 10.7759/cureus.108323

**Published:** 2026-05-05

**Authors:** Nneoma Uzoukwu, Enrique Hernandez, Israh Akhtar, Jeremy Price

**Affiliations:** 1 Obstetrics, Gynecology, and Reproductive Sciences, Lewis Katz School of Medicine at Temple University, Philadelphia, USA; 2 Pathology and Laboratory Medicine, Temple University Hospital, Philadelphia, USA; 3 Radiation Oncology, Temple University Hospital, Philadelphia, USA

**Keywords:** endosalpingiosis, high-grade serous carcinoma, nodal disease, radiation therapy, unknown primary

## Abstract

High-grade serous carcinoma (HGSC) is an aggressive malignancy that may arise from the endometrium, ovary, fallopian tube, or peritoneum, but in rare cases, the primary site remains unknown. Isolated nodal presentation is particularly uncommon, and in such cases, it is inferred to be metastasis. Serous carcinoma of unknown primary (SCUP) lacks management guidelines, with treatment often extrapolated from other gynecologic cancers. We describe the case of a woman with isolated inguinal and pelvic nodal SCUP who achieved durable disease control with definitive radiotherapy after progression on endocrine therapy.

An 88-year-old woman presented with a large left inguinal mass. Biopsy demonstrated HGSC of Müllerian origin. She was initiated on tamoxifen 20 mg twice a day; however, after seven months, the inguinal mass enlarged, and CA-125 levels increased from 2,691 U/mL to 4,191 U/mL. Given her frailty and the morbidity associated with resecting nodes with clinical extracapsular extension, the patient underwent definitive external beam radiotherapy (55 Gy/25 fractions to involved nodes; 45 Gy/25 fractions to the ipsilateral groin and hemipelvis). Six months after radiotherapy, CT showed a reduction of the inguinal mass and pelvic sidewall lymph node, with CA-125 levels declining to 19 U/mL. The patient has remained without disease progression by imaging and CA-125 for 30 months following completion of radiotherapy.

This case describes an uncommon presentation of HGSC of Mullerian origin with isolated inguinal nodes in the absence of identifiable uterine, adnexal, or peritoneal primary. In the absence of established guidelines, it demonstrates the feasibility of definitive radiotherapy in achieving durable disease control.

## Introduction

High-grade serous carcinoma (HGSC) represents the most common subtype of epithelial ovarian carcinomas and comprises 5%-10% of endometrial cancers [1]. It is an aggressive malignancy that may arise from the endometrium, ovary, fallopian tube, or peritoneum; however, in rare instances, the primary site remains occult. In such cases, the disease is classified as serous carcinoma of unknown primary.

Isolated nodal involvement without evidence of a uterine or adnexal primary is exceedingly rare and remains poorly characterized. The absence of a detectable primary tumor creates significant diagnostic challenges and complicates management decisions, as no standardized treatment approach has been established. Only a limited number of case reports have described serous carcinoma confined to lymph nodes, and the pathogenesis remains unclear [2-4]. An emerging theory has proposed that these tumors may arise through de novo malignant transformation from benign Müllerian inclusions such as endosalpingiosis [3].

Endosalpingiosis is characterized by the presence of benign, tubal-type epithelium in ectopic locations such as the peritoneum, omentum, or lymph nodes. Recent literature has demonstrated that endosalpingiosis is more prevalent than previously recognized and has further supported the hypothesis that these benign inclusions may serve as precursor lesions with a potential for neoplastic transformation [5,6]. Several studies have reinforced this hypothesis by revealing a close relationship between endosalpingiosis and the development of serous tumors [3].

We report a case of HGSC of Müllerian origin presenting with isolated inguinal and pelvic nodal disease in a patient without identifiable uterine or adnexal involvement. The patient’s disease progressed on hormonal therapy but responded to definitive radiotherapy, highlighting the feasibility of radiotherapy as a definitive treatment option in select patients.

“This article was previously presented as a poster presentation at the ACOG 2025 District III Junior Fellows Day on October 10, 2025.”

## Case presentation

An 88-year-old woman with a medical history significant for aortic stenosis, chronic atrial fibrillation, pulmonary hypertension, tricuspid and mitral regurgitation, hypertension, and hyperlipidemia presented with a large, painful, slowly progressive inguinal mass. She denied postmenopausal bleeding, fever, night sweats, or unintentional weight loss. Her medical history also included a prior cholecystectomy. Baseline imaging demonstrated mild hepatosplenomegaly, hepatic steatosis, and cardiomegaly with severe vascular calcification of the abdominal aorta and major branches. Laboratory studies at the time of presentation were unremarkable, and she had no pertinent family history. Retrospective review of a CT abdomen and pelvis performed seven years prior revealed mild left inguinal adenopathy.

At the time of presentation, physical examination revealed a fixed, firm, non-tender left inguinal mass. Focused ultrasound revealed a heterogeneous, solid mass measuring 9 cm. Pelvic ultrasound demonstrated a small, unremarkable uterus with no adnexal abnormalities. Two months later, the patient underwent a CT scan of the chest, abdomen, and pelvis. The scan revealed a left inguinal lymph node conglomerate with evidence of necrosis and calcification. An enlarged left pelvic sidewall lymph node was also identified. There was no right-sided pelvic involvement or evidence of primary uterine, adnexal, or peritoneal lesion, supporting isolated nodal involvement.

**Figure 1:**
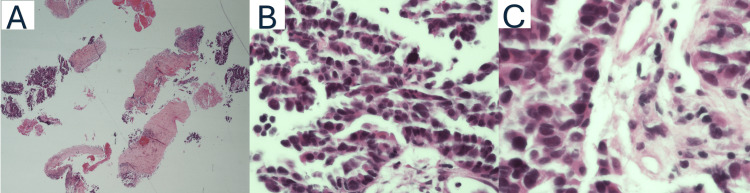
Histopathologic features of high-grade serous carcinoma in a left inguinal lymph node (A) Left Inguinal lymph node core biopsy demonstrating infiltrative tumor fragments (2x). (B, C) High-grade serous carcinoma showing marked nuclear pleomorphism (>3-fold variation in nuclear size) and severe nuclear atypia (40x and 60x, respectively).

**Figure 2:**
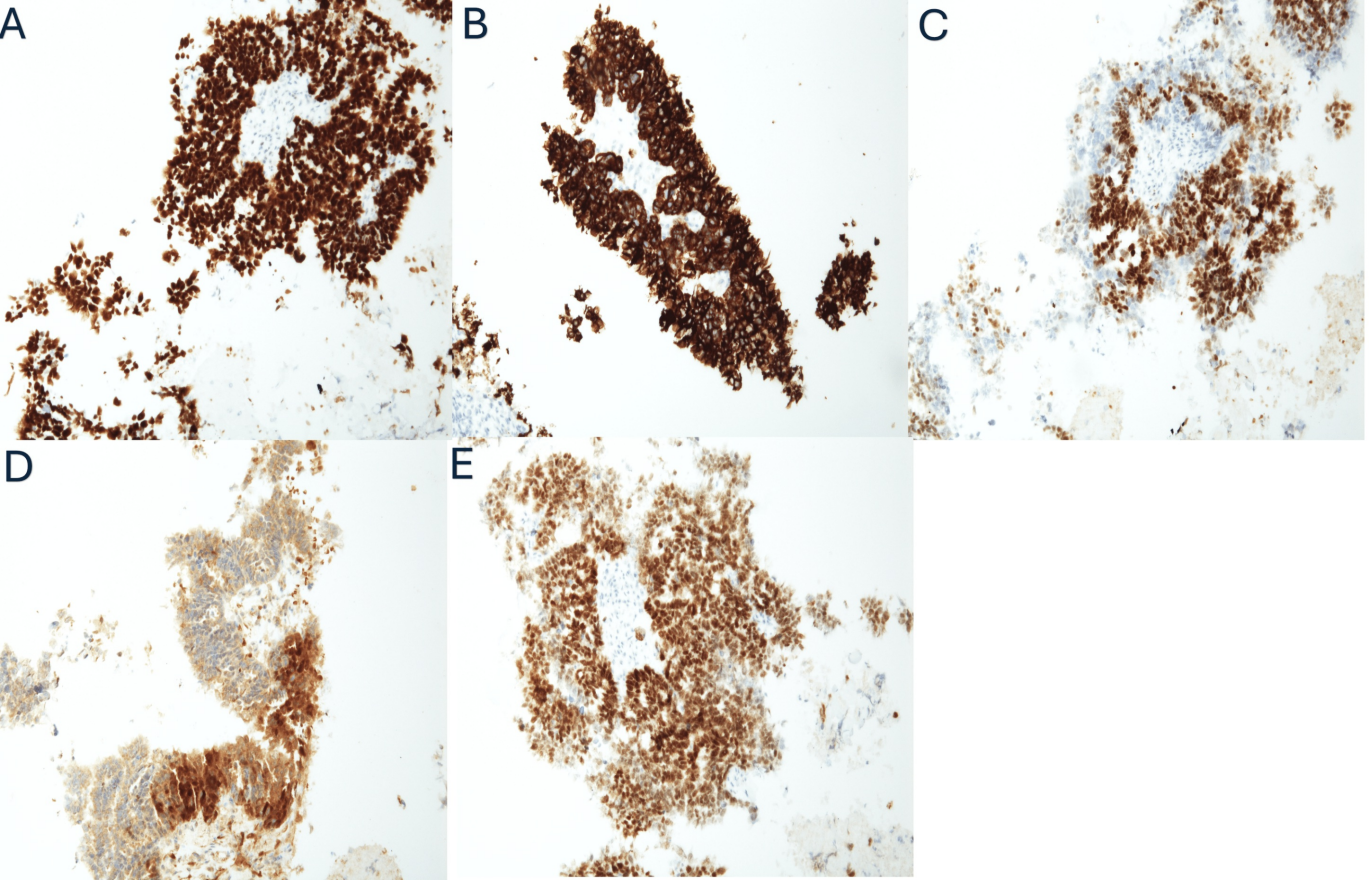
Immunohistochemical characterization of malignant cells (A) Strong and diffuse aberrant nuclear p53 staining, consistent with TP53 missense mutation. (B) Strong and diffuse membranous staining for CK7. (C) Nuclear positivity for WT1. (D) Patchy p16 staining. (E) Strong and diffuse nuclear staining for PAX8, supporting Müllerian origin.

A core biopsy of the left inguinal mass was performed, with pathology demonstrating HGSC of Mullerian origin (Figure 1). Immunohistochemistry demonstrated tumor cells positive for CK7, Pax-8, p53 (diffuse, strong), WT-1, p16 (patchy), and estrogen receptor (ER), while negative for CK20, CDX2, and TTF-1 (Figure 2). No foci of endosalpingiosis were identified in the biopsy specimen. Laboratory tests revealed an elevated cancer antigen (CA-125) level of 2,691 U/mL. Given the patient’s frailty, extensive cardiac comorbidities, and the morbidity associated with resecting lymph nodes demonstrating extracapsular extension, surgical management was deferred in favor of a non-surgical approach. Non-surgical management with endocrine therapy was initiated, and the patient was started on tamoxifen 20 mg twice a day.

**Figure 3:**
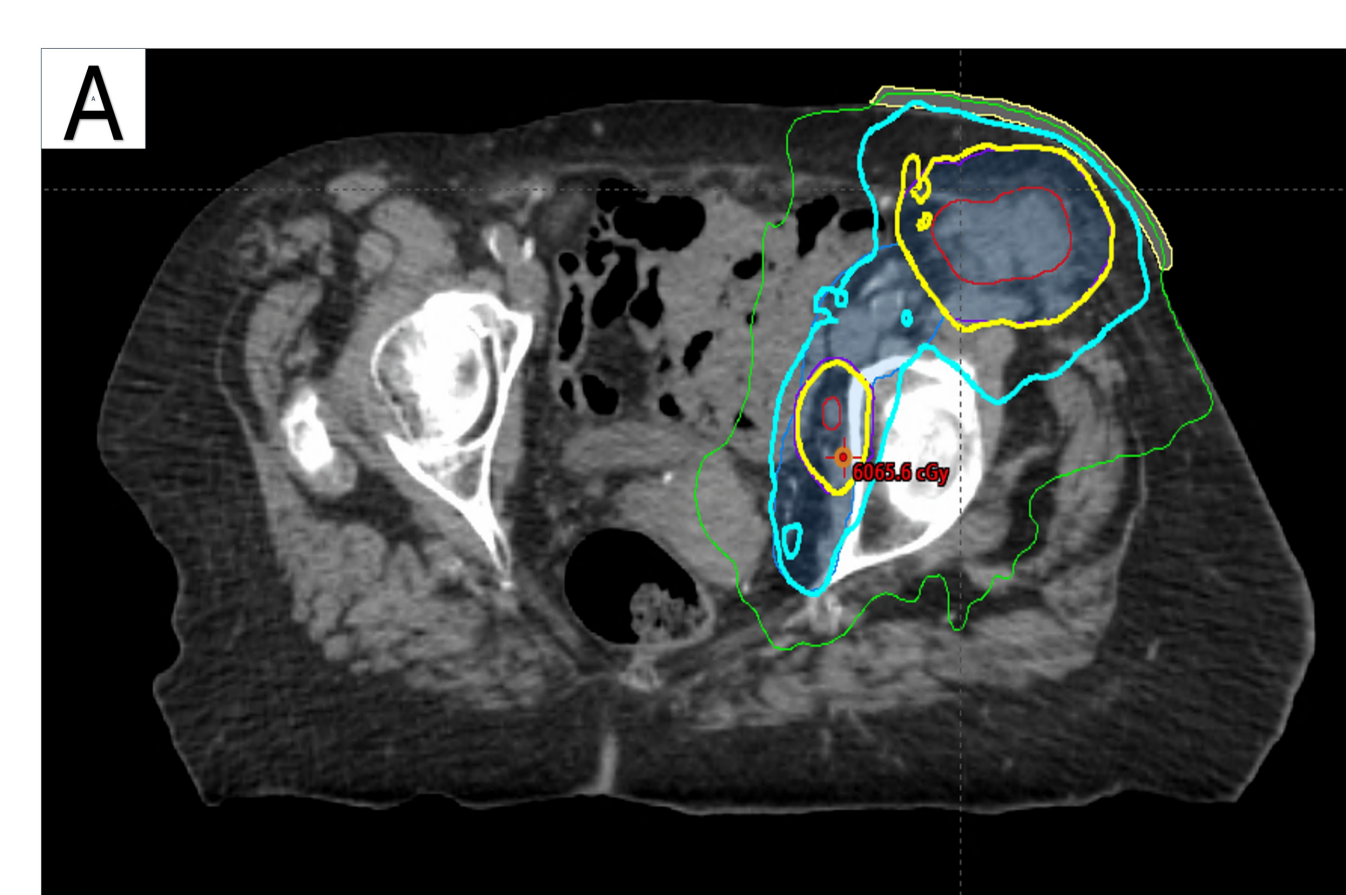
Radiation therapy field illustrating dose distribution and target coverage (A) Representative axial CT image of volumetric modulated arc therapy simultaneous integrated boost plan targeting the lymph nodes and the ipsilateral groin and hemipelvis. The red contour outlines the gross tumor volume, and the blue shaded region represents the planning target volume receiving 45 Gy. The cyan and yellow lines denote the 45 Gy and 55 Gy dose levels, respectively.

**Figure 4:**
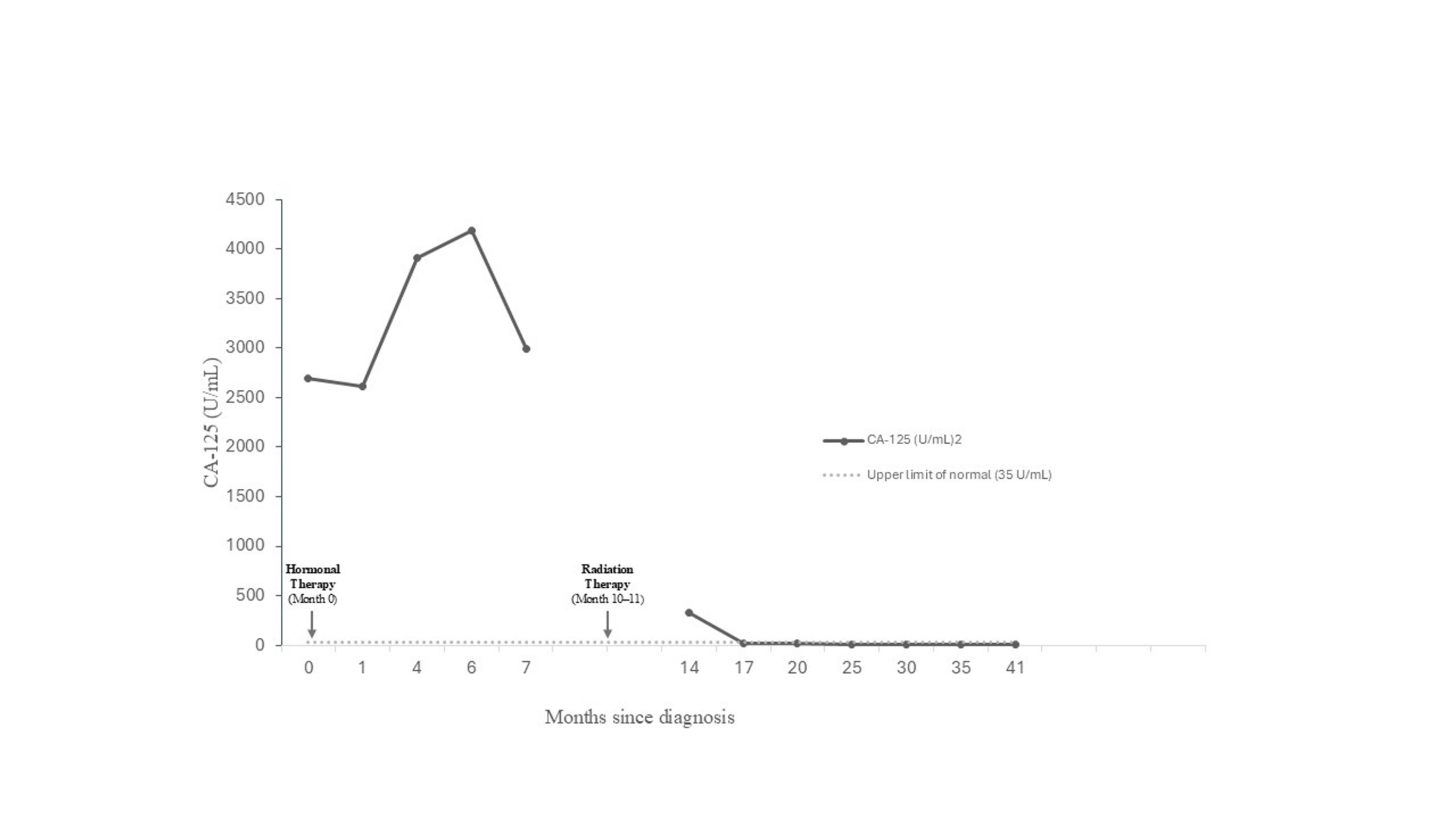
CA-125 levels since diagnosis Values peaked at 4,191 U/mL at month 6 during hormonal therapy, followed by a sustained decline after initiation of radiation therapy (months 10 and 11). Levels normalized by month 17. CA-125 levels were not measured between months 7 and 14. The upper limit of normal is 35 U/mL.

**Figure 5:**
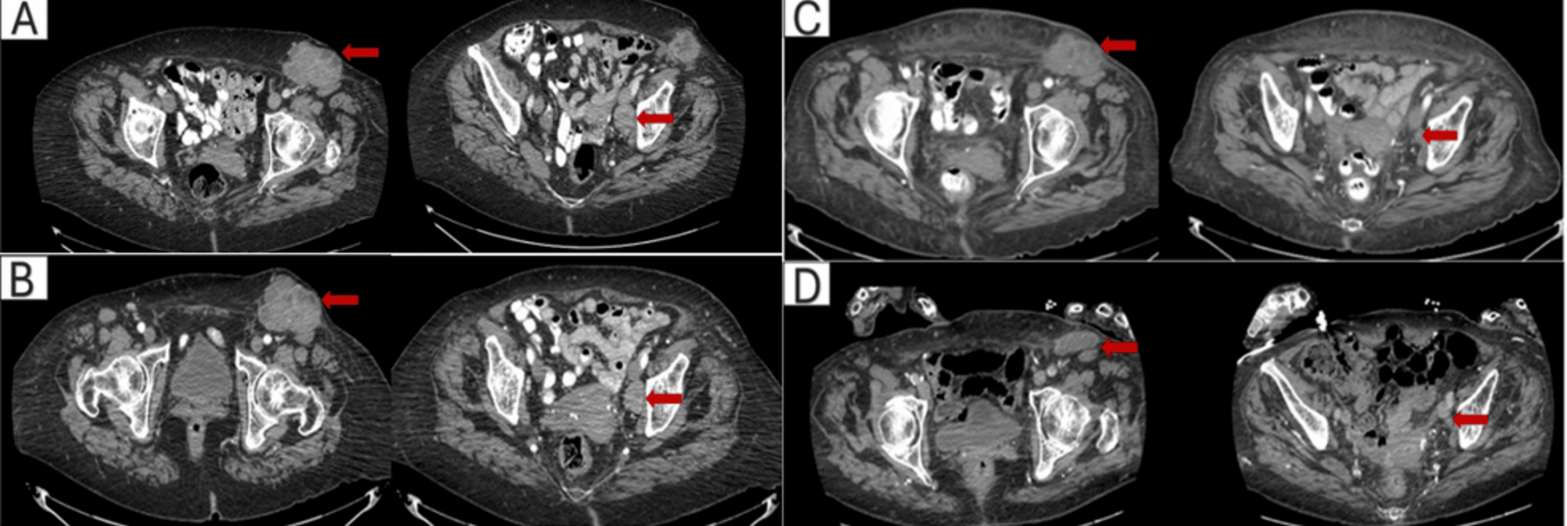
Difference in size of inguinal lymph nodes over course of treatment Axial CT images of the pelvis (A-D) demonstrating progressive reduction in left inguinal lymphadenopathy during treatment. Red arrows indicate representative nodes. (A) Baseline at diagnosis. (B) Eight months after diagnosis. (C) 21 months after diagnosis. (D) 40 months after diagnosis, showing residual nodes. Hormonal therapy was initiated at baseline, and radiation therapy was administered between months 10 and 11.

Unfortunately, despite seven months of treatment, CA-125 levels progressively increased, peaking at 4,191 U/mL. Furthermore, a follow-up CT abdomen/pelvis showed an increase in inguinal mass size. PET showed multiple hypermetabolic left inguinal nodes and a left pelvic wall lymph node. There were no other areas of abnormal fluorodeoxyglucose uptake. The patient was referred to radiation oncology for consideration of either definitive or palliative treatment. Following consultation, the treating radiation oncologist recommended a five-week course of external beam radiation therapy, consisting of a simultaneous integrated boost delivering 55 Gy in 25 daily fractions to the involved lymph nodes and 45 Gy in 25 fractions to the ipsilateral groin and hemipelvis (Figure 3). Subsequent to treatment, marked decline in CA-125 was noted, ultimately reaching a nadir of 19 U/mL (Figure 4). Following radiotherapy, CT imaging demonstrated an interval change in lymph node size. At diagnosis, the nodes measured 67 × 46 mm, 50 × 69 mm, 27 × 35 mm, and 11 × 15 mm (Figure 5A). After eight months of hormonal therapy, several nodes increased in size to 72 × 70 mm, 63 × 62 mm, 30 × 22 mm, and 14 × 10 mm (Figure 5B). By 21 months, these nodes had decreased substantially to 58 × 49 mm and 10 × 15 mm (Figure 5C), and at 40 months, only a single 51 × 18 mm residual node remained (Figure 5D).

The patient continued tamoxifen as a preventive measure since the cancer was ER-positive. She tolerated the course of radiotherapy well, with no significant acute toxicities and no change in baseline performance status. She underwent serial CT scans for oncologic surveillance. Follow-up imaging at 10 and 19 months after completion of radiation therapy demonstrated a stepwise decrease in size of the treated nodes with sustained normalization of CA-125 levels, consistent with durable disease control.

## Discussion

This case highlights an unusual presentation of HGSC of Müllerian origin with isolated inguinal nodes in the absence of identifiable uterine, adnexal, or peritoneal primary. HGSC of the endometrium typically follows an aggressive course, with frequent peritoneal spread, adnexal invasion, and distant dissemination [7]. Nodal-only presentations of HGSC are rarely encountered, and no standardized management has been established [4]. In our patient, the disease progressed on hormonal therapy but demonstrated improvement following definitive radiotherapy, highlighting the feasibility of radiotherapy in achieving durable disease control in selected cases.

Management strategies and treatment outcomes for nodal-only HGSC reported in the literature vary widely. The absence of a detectable primary tumor creates diagnostic challenges and requires extensive histological and immunohistochemical evaluation to confirm Müllerian origin and exclude other primary sites. Restaino et al also describe a patient with a similar presentation to ours who achieved only a temporary response to carboplatin-paclitaxel chemotherapy, with recurrence at 11 months in the contralateral inguinal lymph node [4]. The patient subsequently received pegylated liposomal doxorubicin, which failed, and was ultimately treated with radiotherapy. In contrast, Dam et al. reported complete remission in a patient with nodal disease treated with three cycles of carboplatin-paclitaxel-bevacizumab [8].

For HGSC, the American Society of Clinical Oncology recommends a multimodal approach consisting of comprehensive surgical staging followed by chemotherapy due to its high risk of recurrence [9]. The standard chemotherapy generally involves using carboplatin (AUC 5-6) and paclitaxel (175 mg/m²) every three weeks for six cycles. However, surgery and systemic therapy may be poorly tolerated in elderly or medically frail patients [10]. Our patient was too frail for surgery, and chemotherapy was omitted due to anticipated poor tolerance. Endocrine therapy with tamoxifen was initiated based on the tumor’s ER positivity. Although hormonal therapy is not typically standard in HGSC, retrospective data suggest potential benefit in ER-positive disease, particularly among patients who are poor candidates for chemotherapy [11].

Despite initial endocrine management, the patient’s disease progressed on tamoxifen, with enlarging nodal size and rising CA-125 levels, necessitating an alternative treatment strategy. We pursued definitive radiotherapy modeled after the EMBRACE protocol: 55 Gy (60 Gy EQD2) was delivered to the involved nodes via simultaneous integrated boost, while 45 Gy to the at-risk hemipelvis in 25 fractions [12]. Following radiotherapy, CA-125 levels normalized, and imaging demonstrated a marked reduction in nodal size.

Since no clear primary site was identified, we hypothesize that the carcinoma may have arisen de novo from Müllerian inclusions, such as endosalpingiosis within a lymph node, rather than representing metastasis from an occult primary. The absence of identifiable endosalpingiosis in the limited biopsy specimen does not exclude its presence. Endosalpingiosis is a benign condition characterized by the presence of ectopic tubal-type epithelium [2]. It is typically found in the peritoneum and omentum but may also be found within pelvic and para-aortic lymph nodes. The pathogenesis remains unclear, though it is generally thought to result from displaced embryonic Müllerian tissue, a process termed Müllerianosis [2].

Although endosalpingiosis is typically considered an incidental histological finding, emerging literature suggests that it may be far more prevalent and potentially more clinically significant than previously recognized. In a retrospective review of approximately 60,000 patients who underwent gynecologic surgeries between 1998 and 2013, Esselen et al. found 838 (1.4%) cases of pathologically confirmed endosalpingiosis. Among these cases, 42% also had a gynecologic malignancy, compared with 23% in women without endosalpingiosis [5]. To further expand on this relationship, Sunde et al. utilized a more comprehensive protocol for examining the fallopian tubes and adnexa (SEE-Fim) in a retrospective analysis of approximately 600 gynecologic surgical specimens [6]. Endosalpingiosis was present in 22% (p<0.001) of cases with a benign-appearing adnexal mass, with prevalence rising to 28% (p<0.001) in women over 30 years and to 66% in postmenopausal women. Among postmenopausal patients with gynecologic malignancy, 74% also had endosalpingiosis. Djordjevic et al. further reported that nodal ES was significantly more common in ovarian serous tumors of low malignant potential with nodal involvement (66%) than those without nodal involvement (14%, p<0.0001) [3].

Furthermore, Sah et al. describe two cases of low-grade serous carcinoma confined to inguinal nodes and noted that there have been only three prior reports of similar presentation [13]. In both cases, endosalpingiosis was present in or adjacent to involved nodes, supporting the hypothesis of a local malignant transformation rather than metastasis from an occult primary. They proposed that some nodal low-malignant foci may arise de novo from pre-existing endosalpingiosis, a mechanism that may also explain our patient’s presentation.

## Conclusions

Recognition of nodal endosalpingiosis as a potential precursor is critical not only to avoid misdiagnosis and unnecessary overtreatment but also to acknowledge its rare yet important potential for malignant transformation. Ultimately, our case adds to the limited literature on nodal-only Müllerian carcinoma and contributes to the growing recognition that these lesions may not always represent metastasis from an occult primary but instead arise directly from inclusions within lymph nodes. This has important implications not only for diagnosis but also for management. In our patient, definitive radiotherapy provided durable disease control in the absence of surgery or systemic therapy, underscoring its feasibility as a treatment strategy in select patients.

## References

[1] Kim J, Park EY, Kim O, Schilder JM, Coffey DM, Cho CH, Bast RC Jr (2018). Cell origins of high-grade serous ovarian cancer. Cancers.

[2] Solaru SA, Liu MC, Lee V, Bristow RE (2025). Isolated low-grade serous carcinoma arising in inguinal lymph nodes in the setting of endosalpingiosis: a case report. Gynecol Oncol Rep.

[3] Djordjevic B, Clement-Kruzel S, Atkinson NE, Malpica A (2010). Nodal endosalpingiosis in ovarian serous tumors of low malignant potential with lymph node involvement: a case for a precursor lesion. Am J Surg Pathol.

[4] Restaino S, Mauro J, Zermano S (2022). CUP-syndrome: inguinal high grade serous ovarian carcinoma lymph node metastases with unknown primary origin - a case report and literature review. Front Oncol.

[5] Esselen KM, Terry KL, Samuel A (2016). Endosalpingiosis: more than just an incidental finding at the time of gynecologic surgery?. Gynecol Oncol.

[6] Sunde J, Wasickanin M, Katz TA, Wickersham EL, Steed DO, Simper N (2020). Prevalence of endosalpingiosis and other benign gynecologic lesions. PLoS One.

[7] Bogani G, Ray-Coquard I, Concin N (2021). Uterine serous carcinoma. Gynecol Oncol.

[8] Dam K, Peeters F, Verhoeven D, Duwel V (2021). High-grade serous cancer of undetermined primary origin presenting as solitary inguinal lymph node enlargement. BMJ Case Rep.

[9] Gaillard S, Lacchetti C, Armstrong DK (2025). Neoadjuvant chemotherapy for newly diagnosed, advanced ovarian cancer: ASCO guideline update. J Clin Oncol.

[10] Dale W, Klepin HD, Williams GR (2023). Practical assessment and management of vulnerabilities in older patients receiving systemic cancer therapy: ASCO guideline update. J Clin Oncol.

[11] Stanley B, Hollis RL, Nunes H (2019). Endocrine treatment of high grade serous ovarian carcinoma; quantification of efficacy and identification of response predictors. Gynecol Oncol.

[12] Pötter R, Tanderup K, Kirisits C (2018). The EMBRACE II study: the outcome and prospect of two decades of evolution within the GEC-ESTRO GYN working group and the EMBRACE studies. Clin Transl Radiat Oncol.

[13] Sah S, Fulmali R, McCluggage WG (2020). Low-grade serous carcinoma arising in inguinal nodal endosalpingiosis: report of 2 cases and literature review. Int J Gynecol Pathol.

